# Pulsed thulium:YAG laser—ready to dust all urinary stone composition types? Results from a PEARLS analysis

**DOI:** 10.1007/s00345-023-04549-y

**Published:** 2023-08-16

**Authors:** Jia-Lun Kwok, Eugenio Ventimiglia, Vincent De Coninck, Mariela Corrales, Alba Sierra, Frédéric Panthier, Felipe Pauchard, Florian Schmid, Manuela Hunziker, Cédric Poyet, Michel Daudon, Olivier Traxer, Daniel Eberli, Etienne Xavier Keller

**Affiliations:** 1 Department of Urology, University Hospital Zurich, University of Zurich, Frauenklinikstrasse 10, 8091 Zurich, Switzerland; 2 Department of Urology, Tan Tock Seng Hospital, Singapore, Singapore; 3 Progressive Endourological Association for Research and Leading Solutions (PEARLS), Paris, France; 4 Young Academic Urologists (YAU), Endourology and Urolithiasis Working Group, Arnhem, The Netherlands; 5 Division of Experimental Oncology/Unit of Urology, Urological Research Institute, IRCCS Ospedale San Raffaele, Milan, Italy; 6 Department of Urology, AZ Klina, Brasschaat, Belgium; 7 GRC n°20, Groupe de Recherche Clinique sur la Lithiase Urinaire, Hôpital Tenon, Sorbonne Université, 75020 Paris, France; 8 Urology Department, Hospital Clinic de Barcelona, Villarroel 170, 08036 Barcelona, Spain; 9 Urology Department, Hospital Naval Almirante Nef, 2520000 Viña del Mar, Chile; 10 CRISTAL Laboratory, Hôpital Tenon, Sorbonne Université, Paris, France

**Keywords:** Laser, Pulsed thulium:YAG, Kidney stones, Stone dust, Endourology, Ureteroscopy

## Abstract

**Purpose:**

To evaluate whether stone dust can be obtained from all prevailing stone composition types using the novel pulsed thulium:YAG (p-Tm:YAG), including analysis of stone particle size after lithotripsy.

**Methods:**

Human urinary stones of 7 different compositions were subjected to in vitro lithotripsy using a p-Tm:YAG laser with 270 µm silica core fibers (Thulio^®^, Dornier MedTech GmbH^®^, Wessling, Germany). A cumulative energy of 1000 J was applied to each stone using one of three laser settings: 0.1 J × 100 Hz, 0.4 J × 25 Hz and 2.0 J × 5 Hz (average power 10 W). After lithotripsy, larger remnant fragments were separated from stone dust using a previously described method depending on the floating ability of dust particles. Fragments and dust samples were then passed through laboratory sieves to evaluate stone particle count according to a semiquantitative analysis relying on a previous definition of stone dust (i.e., stone particles ≤ 250 µm).

**Results:**

The p-Tm:YAG laser was able to produce stone dust from lithotripsy up to measured smallest mesh size of 63 µm in all seven stone composition types. Notably, all dust samples from all seven stone types and with all three laser settings had high counts of particles in the size range agreeing with the definition stone dust, i.e., ≤ 250 µm.

**Conclusion:**

This is the first study in the literature proving the p-Tm:YAG laser capable of dusting all prevailing human urinary stone compositions, with production of dust particles ≤ 250 µm. These findings are pivotal for the broader future implementation of the p-Tm:YAG in clinical routine.

## Introduction

Urinary stone disease has become increasingly prevalent worldwide [1–5]. For more than three decades, the laser has been established as the mainstay of treatment when it comes to endourological surgery and lithotripsy [6]. Particularly over the last decade, dusting properties of lasers used for lithotripsy have become increasingly recognized as an important factor affecting major outcomes of stone surgery [7–9].

Currently, the holmium:yttrium–aluminum–garnet (Ho:YAG) and the more recent thulium fiber laser (TFL) are widely used in endourological procedures [10–12]. Additionally, novel pulsed thulium:yttrium–aluminum–garnet (p-Tm:YAG) lasers have recently been introduced to the market for clinical use. Based on a limited series of in vitro evaluations, the p-Tm:YAG laser seems to come with promising stone dusting properties [13, 14]. It is important to note that these preliminary reports were based on lithotripsy models using artificial Bego Stones—and not human urinary stones—with the primary outcome being stone ablation efficiency rather than evaluation of stone dust per se. Two recent in vivo studies evaluated the clinical efficacy and safety of the p-Tm:YAG on case series of patients undergoing retrograde intrarenal surgery [15] and mini-percutaneous nephrolithotomy [16]. Both studies conclude that the p-Tm:YAG seems very promising, with patients included having a range of stone densities, although no information regarding exact differing stone compositions was provided. In the mini-percutaneous nephrolithotomy study, the authors specifically state not investigating stone composition as a study limitation. Considering the above, a study is warranted to verify whether the new p-Tm:YAG laser is capable of dusting prevailing human urinary stone types. For this purpose, the present study includes an established lithotripsy and stone dust collection model, with analysis of stone particles size after lithotripsy of the seven most common human urinary stone types using the new p-Tm:YAG laser.

## Material and methods

Human urinary stones were retrieved from a large stone biobank from Tenon Hospital, Paris encompassing the following stone composition types: calcium oxalate monohydrate (COM), calcium oxalate dihydrate (COD), uric acid (UA), carbapatite (CA), struvite (STR), brushite (BR) and cystine (CYS). Stones were selected to match a volume of approximately 100 to 200 mm^3, were extracted without laser lithotripsy and had a > 90% degree of purity based on infrared spectroscopy. To simulate in vivo settings, all stones were submerged in saline for 24 h prior to experiments, as kidney stones are of a crystalline structure primarily, but grow in a biological environment with complex intercrystalline spaces [17] likely filled with urine.

Each stone was separately subjected to laser lithotripsy using the Dornier^®^ Thulio^®^ p-Tm:YAG with its 270-µm-core-diameter Dornier^®^ Thulio^®^ Performance reusable laser fiber (Dornier MedTech GmbH^®^, Wessling, Germany). The characteristics of the novel p-Tm:YAG laser are summarized in Table 1.

**Table 1:** Pulsed thulium:YAG (p-Tm:YAG) laser characteristics^a^

Characteristic		Description		
Laser energy source		Solid-state, diode-pumped, thulium-doped YAG crystal		
Wavelength		2013 nm		
Laser settings	Operating mode	Pulsed only		
	Output power	100W		
	Peak power	Max 3.7 kW		
	Pulse energy range	0.1–2.5 J		
	Pulse frequency range	5–300 Hz		
	Pulse length range	150–1200 μs		
Laser fibers	Laser fibers dimensions	Manufacturer label	Core diameter	Outer diameter
		270 Micron Slim	272 ± 5 µm	400 ± 30 µm
		400 Micron	365 ± 10 µm	550 ± 30 µm
		600 Micron	550 ± 12 µm	750 ± 30 µm
		1000 Micron	940 ± 15 µm	1400 ± 50 µm
	Length of laser fiber	3 m		
	Reusability of laser fiber	Single use and reusable (10x) available in same sizes		
Machine	Machine weight	97 kg (laser device)		
	Machine dimensions	(W) 42 × (D) 62 × (H) 139 cm (Including monitor)		
	Operating noise emission	≤ 65 dB		
	Power source requirement	115/208–240 VAC, single phase, max. 15 A, 50/60 Hz		
	Cooling system	Internal closed-loop water cooling system		
	Pedal characteristics	Two-pedal footswitch with two additional buttons—(Standby/Ready and changing of laser settings). Wireless and wired options		

^a^According to manufacturer

We used three laser settings for lithotripsy: 0.1 J × 100 Hz, 0.4 J × 25 Hz and 2.0 J × 5 Hz, with each sample from the same stone type being treated with a different laser setting and reaching a cumulative applied energy of 1000 J.

All settings resulted in an average power of 10W. For the first setting, we chose the lowest pulse energy available (0.1 J) on the graphical user interface (GUI) touchscreen in “Dusting” mode with an according frequency (100 Hz) to reach 10W. For the second setting, we chose a pulse energy that is generally accepted as an adequate dusting setting (0.4 J × 25 Hz) for Ho:YAG and TFL lithotripsy (“Dusting mode” on the GUI) [9, 18]. For the third setting, 2.0 J × 5 Hz was chosen as a typical fragmenting setting, meeting the aforementioned 10 W agreement (“Standard Fragmenting” mode on the GUI). Since pulse duration could not be changed and was not displayed in the “Dusting” and “Standard Fragmenting” modes, we did not explore this laser setting. For each stone sample, as the average power used (10 W) was the same to reach a common cumulative energy (1000 J), this means the lasing time was the same throughout all experiments (1000 J/10 W = 100 s).

**Fig. 1:**
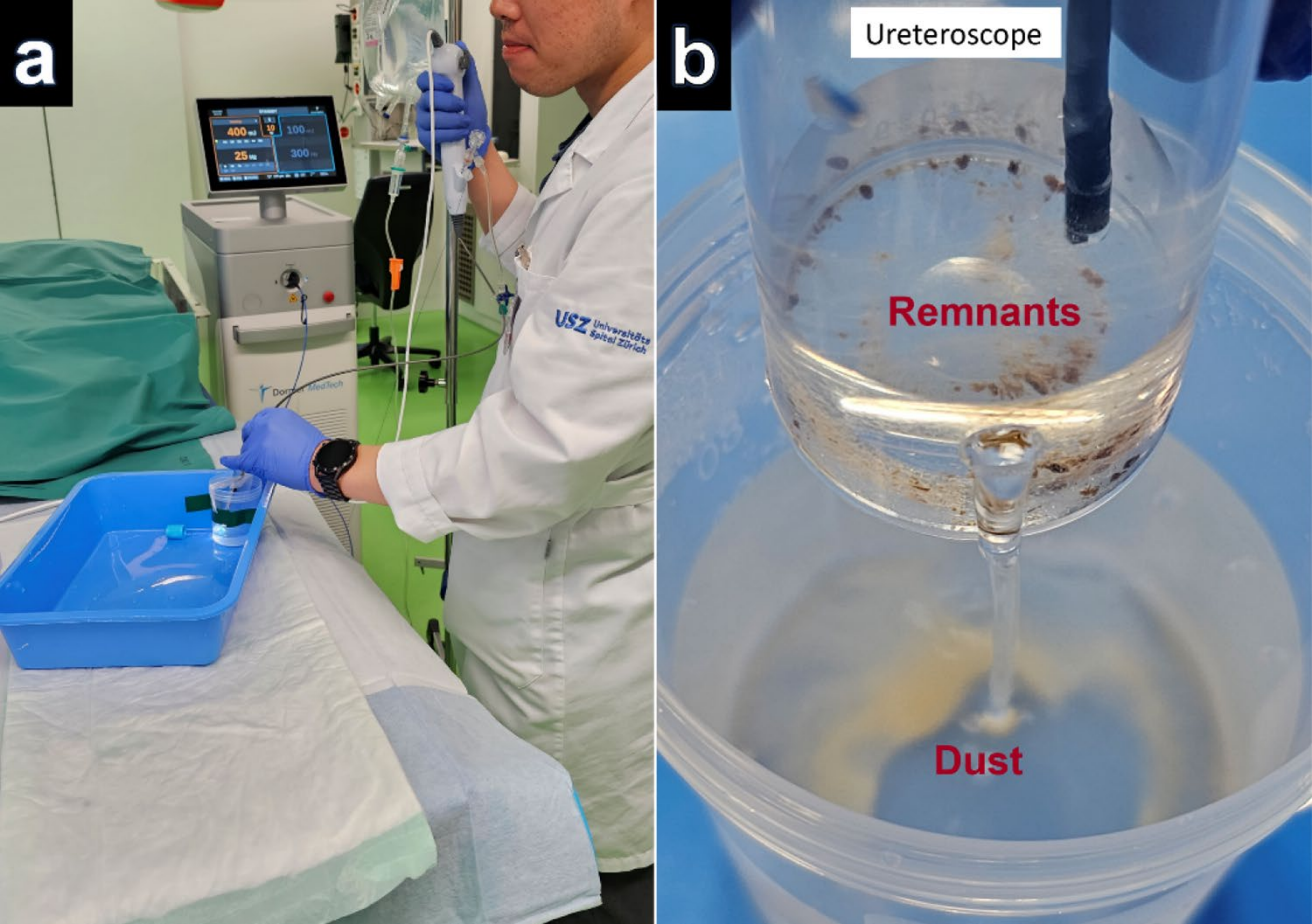
Experimental setup for lithotripsy and separation process. **a** Lithotripsy setup with ureteroscope inserted into a glass cuvette, **b** post-lithotripsy separation process in a “remnants” 60 ml container with a hole at 1 cm from bottom of container and overflow into a “dust” 100 ml container

The OTU WiScope (OTU Medical Inc, CA, USA) flexible ureteroscope was used for laser lithotripsy under direct endoscopic vision in a 10-ml glass cuvette, with 0.9% sodium chloride under gravity irrigation pressure of 40 cmH20 at room temperature (21 °C) (Fig. 1a). The irrigation overflow was collected in a 100 ml plastic container. Lithotripsy was performed freehand with painting movements of the laser fiber tip over stone samples for the dusting settings and pinpoint movements for the fragmenting laser setting. The laser fiber tip was cut through the blue protective jacket with regular metallic surgical scissors before lithotripsy for each sample. All experiments were performed using the same reusable laser fiber.

Considering that there is currently no generally accepted definition of stone dust, stone dust was separated from larger remnant fragments after laser lithotripsy using a previously described method employed in prior studies [19, 20] that depends on the floating ability of dust particles. This was done by evacuation of spontaneously floating stone dust over a 5 mm hole located 1 cm above the bottom of a 60 ml plastic container upon constant irrigation over the flexible ureteroscope (40 cmH2O, empty working channel) (Fig. 1b). The resultant irrigation overflow was collected in the aforementioned 100 ml plastic container, thus merging all stone dust together in a “dust” sample. The remaining stone fragments in the 60 ml container are referred to as “remnants” sample from here on.

**Fig. 2:**
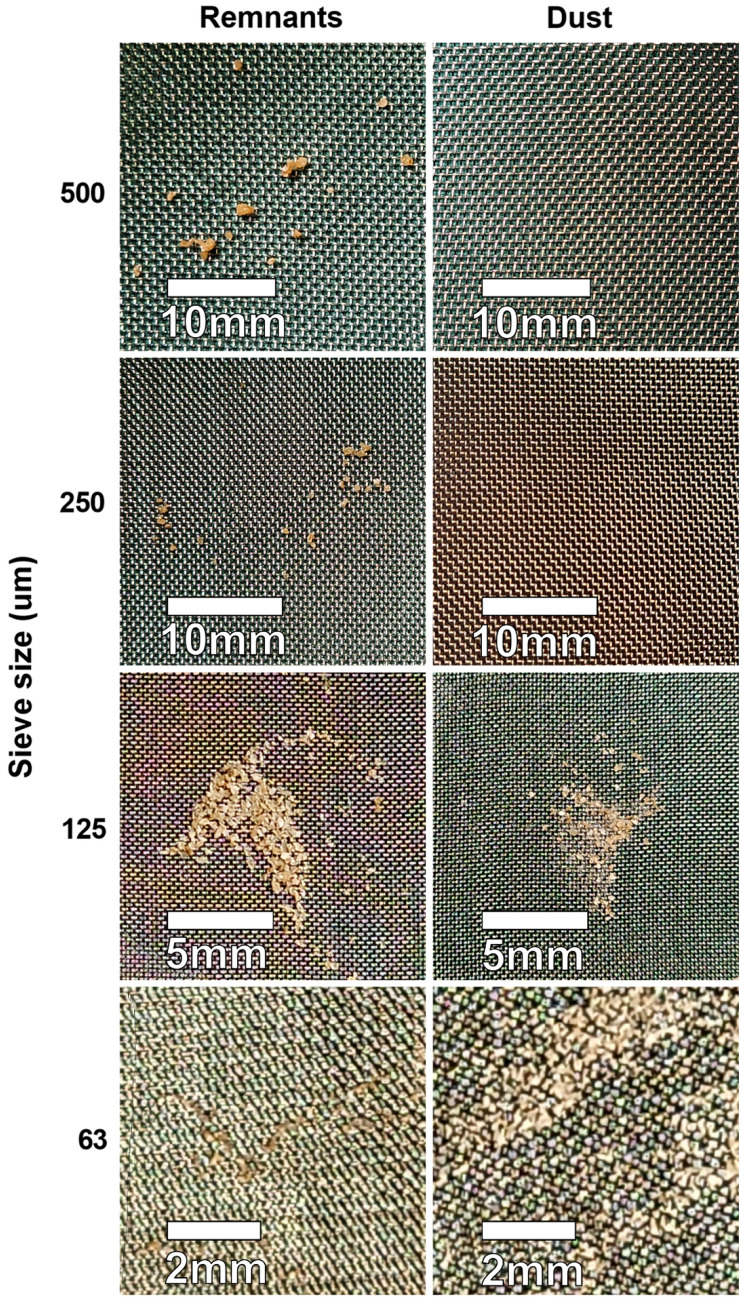
Examples of stone particles gathered on sieves’ surfaces. Images here are cropped parts of the sieve photographs. Particles are from sieving process of a cystine stone subjected to lithotripsy with the p-Tm:YAG laser @ 2.0Jx5Hz = 10W. The semiquantitative analysis of this sample is found in Fig. 3

For stone size analyses, each sample of remnants and dust was then separately further processed by passing through stacked laboratory sieves (Eisco sorting sieves, Eisco Scientific LLC, NY, USA) for semiquantitative analysis (Fig. 2). Mesh size openings of 500 µm, 250 µm, 125 µm and 63 µm were used to separate the stone particles into different size categories. A total of 500 ml of saline was poured over the stacked sieves to ensure full sedimentation of particles over the whole range of sieves. After this, the sieves of each sample were photographed separately for analysis of the stone particle count. To ensure that the whole sieve area was consistently included in the photographs, we used a holder to fix the camera. A reference marker of 1 cm placed on each sieve sample was used to calibrate the scale for Fig. 2 using the software *ImageJ* (version 1.53tRRID:SCR_003070) [21]. A previously proposed stone size limit of ≤ 250 µm was used for the definition of stone dust [7].

### Statistical analysis

The number of stone particles for each sample and each entire sieve surface was evaluated and categorized into particle counts of 1–10 (low), 11–50 (moderate) and > 50 (high) by two authors (JK and EXK), based on the corresponding sieves’ photographs. Discrepancies were resolved by consensus between these two authors. A Spearman’s correlation analysis was performed to compare overall sieve size and respective stone particle count categories. A two-sided *p* value < 0.05 was considered statistically significant. All descriptive and statistical analyses were performed with GraphPad Prism 9.5.1 (GraphPad Software, La Jolla CA, USA).

## Results

**Fig. 3:**
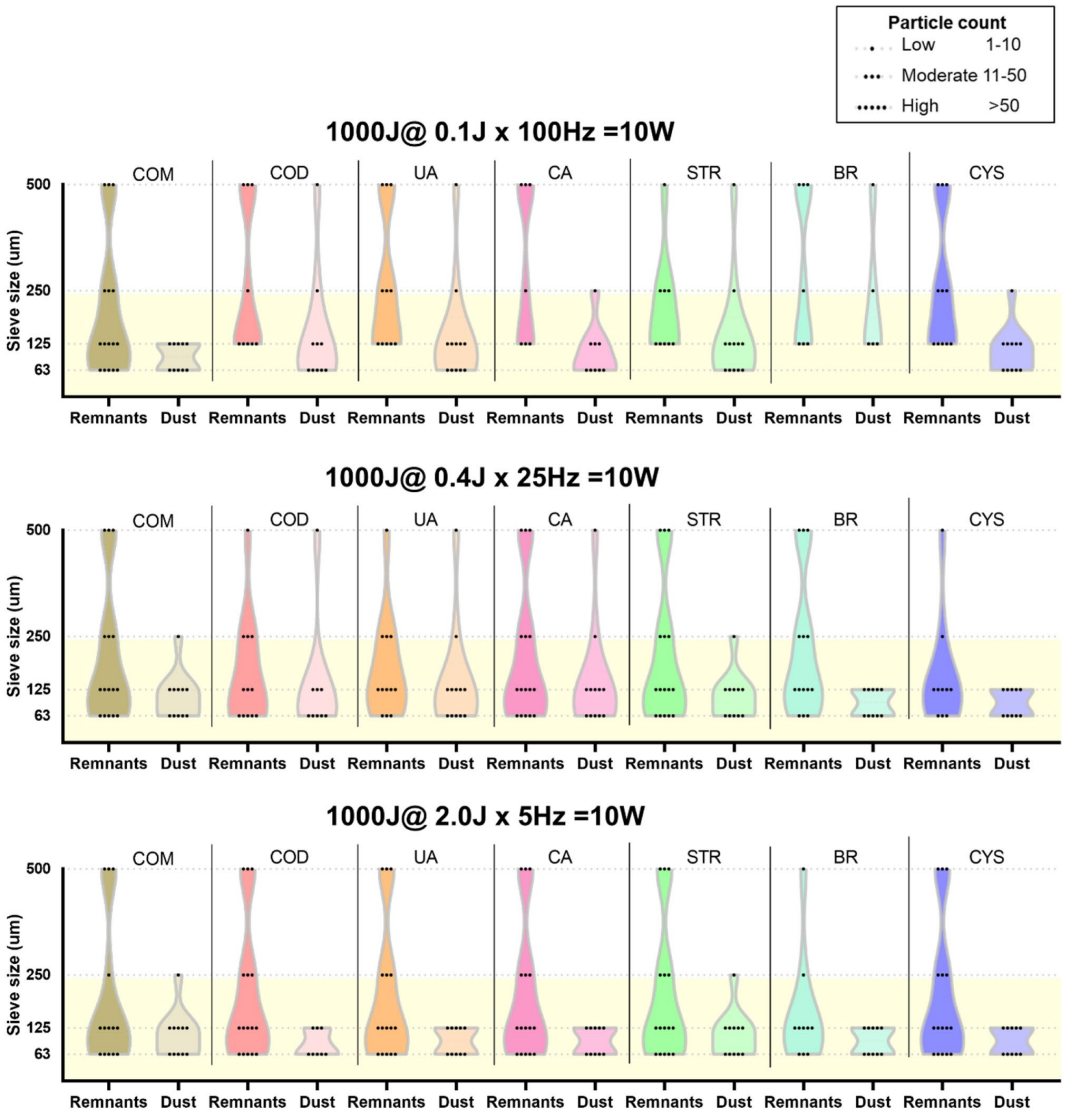
Stone particle size distribution after lithotripsy with p-Tm:YAG. Particle size distribution represented by particle category counts (low, moderate and high) on sieve surfaces of various mesh sizes for three laser settings. Stone compositions: calcium oxalate monohydrate (COM), calcium oxalate dihydrate (COD), uric acid (UA), carbapatite (CA), struvite (STR), brushite (BR) and cystine (CYS). Yellow area denotes the size range from a prior definition of stone dust [7]

The p-Tm:YAG laser was able to produce dust from lithotripsy up to the smallest measured mesh size of 63 µm in all seven urinary stone composition types (Fig. 3).

Particularly, all dust samples from all seven stone types and with all three laser settings were found to have high counts of particles in the size range agreeing with the definition of stone dust, i.e., ≤ 250 µm. A few isolated dust samples showed low count of particles > 250 µm.

In the remnants samples, a low to moderate count of larger stone fragments (> 250 µm) was found in addition to a moderate to high count of stone dust particles (≤ 250 µm) for all seven stone types and with all three laser settings.

When considering comparisons between the different stone types, no obvious pattern of differing stone particle count distribution either for dust or for remnants samples was found within each laser setting. The same was valid when considering comparisons between the different laser settings for each stone type. The only general observation was that the particle count on smaller sieve sizes was consistently higher than on larger sized sieves, resulting in a significant negative correlation when comparing sieve size with stone particle count (*r*_*S*_ = − 0.77, *p* = 0.01).

## Discussion

To the best of our knowledge, this is the first study in the literature to reveal the p-Tm:YAG laser being capable of dusting human urinary stone composition types commonly found in clinical routine. Additionally, this study confirms a size limit of ≤ 250 µm applicable to all stone types for the definition of stone dust, according to a separation process based on the spontaneous floating and evacuation of stone dust [7].

The findings of this study are pivotal for the broader future implementation of the p-Tm:YAG in clinical routine, considering that studies found in the literature so far have presented data based on non-human stone lithotripsy models (BegoStone, plaster of paris, gypsum/glass) [13, 14, 22], or if conducted in humans did not investigate laser effects on different human urinary stone compositions [16]. It is of particular interest to note that the p-Tm:YAG was amenable to lithotripsy of cystine stones, akin to the Ho:YAG and TFL [23]. One may recall the frequency-doubled double-pulse neodymium:YAG (FREDDY) laser which was originally proposed as a potential technology for lithotripsy in the 1980s, until it was found ineffective at fragmenting cystine stones[24, 25]. That shortcoming eventually allowed for the holmium:YAG laser to become the gold standard for lithotripsy in those days. To that extent, the present study falls into the legacy of fundamental research approving newer technology for lithotripsy [20].

Providing a semiquantitative analysis of stone dust is of high importance, considering that stone dusting has become a broadly adopted technique for laser lithotripsy, while the exact definition of stone dust is still a matter a debate. To date, the most comprehensive definition of stone dust relies on a trifecta based on laboratory testing [7]: (1) spontaneous floating with 40 cm H2O irrigation pressure; (2) mean sedimentation time of more than 2 s through 10 cm saline solution; and (3) fully able to be aspirated through a 3.6 F ureteroscope’s working channel. This definition has been applied to in vivo clinical laser studies [26, 27], however clinical relevance has not been fully demonstrated yet.

Small stone particles ≤ 250 µm originating from p-Tm:YAG lithotripsy were found to spontaneously evacuate upon irrigation in the present experimental setup, as prior proved possible with Ho:YAG [19] and TFL studies [20]. These small particles were considered as stone dust per prior stone dust definition [7].

Overall, the smaller the sieve size, the higher the particle count, and vice versa. This significant correlation is arguably explained by the smaller particles being the result of innumerable stone breakdown cycles and the exponential nature of the lithotripsy process (e.g., 1 fragment becomes 16 particles after 4 fragmentation iterations, etc.). The presence of dust particles in the remnants samples is likely due to the fact that the irrigation flow during the separation process was limited to < 100 ml—until the 100 ml container was full. If the separation irrigation process was longer, there would likely be much lower counts of dust in the remnants sample, akin to the ureteroscopic time needed for a retrograde intrarenal surgery procedure or over days post-procedure with the kidney producing urine.

Of interest, we noted the presence of stone dust particles ≤ 250 µm in the 2.0 J × 5 Hz fragmenting setting. This fragmentation setting may in fact translate to a pop-dusting technique, particularly toward the later pulse counts, due to the duration required to apply a total energy of 1000 J in order to maintain consistency of energy applied across all three laser settings in a small glass cuvette [28]. Comparatively, fragmenting lithotripsy techniques would rather rely on a much lower pulse count limited to the breakdown of the initial stone in few fragments for basket extraction. It is still intriguing to see that this high pulse energy setting led to dust in this study, which is an undesirable property for fragmentation purposes, arguably due to potential impairment of vision from produced dust or leading to inefficiency in achieving multiple fragments in as short a possible time. Considering the above, it would be interesting to evaluate which of the current laser technologies is most adequate for fragmentation in future studies.

The authors of the present study want to emphasize that the results found in this study may be inferable to other p-Tm:YAG generators operating at a similar wavelength and with comparable peak powers and pulse durations—although p-Tm:YAG laser generators from differing manufacturers may differ in their properties and shall therefore be evaluated in separate studies. Conversely, the results are not transferable to the TFL, a different laser technology [29] for which a distinct evaluation has been published before [20].

The study has several potential limitations. The present study is an in vitro attempt to assess the p-Tm:YAG laser lithotripsy dusting characteristics that may impact on in vivo surgery. The interpretation of the data must therefore be taken with care since environmental factors arguably may impact on clinical translation of the findings of this study. The size of the initial stones submitted to lithotripsy was rather small and was not standardized; therefore, conclusions on the efficacy of different laser settings cannot be drawn from this study. Rather, the presence and relative number of particles within each stone setting and laser setting was reviewed. Accordingly, stone particle samples were not weighed, but particle numbers counted, and quantified with count categories of different particle sizes, which allowed a semiquantitative analysis of the results. Finally, we noted that there were few large particles of > 250 µm in some dust samples. These few larger particles may have been inadvertently collected as a result of mechanisms such as pop-corning or pop-dusting dynamics during laser lithotripsy and may therefore be understood as sample contaminants that do not impact on the conclusions drawn from this study: The p-Tm:YAG is capable of producing moderate to high amounts of stone dust particles ≤ 250 µm.

Areas for exploration in future studies would include evaluation of stone composition changes with the novel p-Tm:YAG, and investigating mechanisms of relevance for stone dusting that are not perfectly understood yet. It is indeed desirable to have fine stone dust in laser lithotripsy and to refine ways to achieve it.

## Conclusions

The novel p-Tm: YAG laser is capable of dusting all seven common human urinary stone compositions, with the production of dust particles ≤ 250 µm, in keeping with prior definition of stone dust. These findings are pivotal for the broader future implementation of the p-Tm:YAG in clinical routine.

## Data Availability

On request to corresponding author for raw data on the experimental setup.
